# Fallopian Tube Torsion Secondary to Paraovarian Fimbrial Cyst: A Difficult to Diagnose and a Rare Cause of Acute Abdomen in Adolescent

**DOI:** 10.7759/cureus.17888

**Published:** 2021-09-11

**Authors:** Shamail Syed, Ayesha Amin, Muneeb Ullah

**Affiliations:** 1 Obstetrics and Gynecology, Maroof International Hospital, Islamabad, PAK; 2 Radiology, Maroof International Hospital, Islamabad, PAK; 3 Surgery, Maroof International Hospital, Islamabad, PAK

**Keywords:** fimbrial cyst, fallopian tube torsion, paraovarian cyst, acute abdomen, laproscopy

## Abstract

Fallopian tube torsion secondary to paraovarian or paratubal cyst is a rare gynecological cause of acute abdomen. The condition has no distinctive signs and symptoms. There are no characteristic features on radiological imaging, making preoperative diagnosis very difficult. Paraovarian cysts that are less than 4 cm in size are mostly asymptomatic and found incidentally during a pelvic examination or radiological imaging. It seldom leads to any complications like fallopian tube torsion hemorrhage or rupture. We report a case of an adolescent girl, who presented with severe abdominal pain. Transabdominal ultrasound was suggestive of a cystic structure less than 4 cm in size in the left adnexa. Doppler ultrasound showed normal blood flow to both ovaries. Diagnostic laparoscopy was performed, which revealed a twisted left-sided fallopian tube with a fimbrial paraovarian cyst. Detorsion and paraovarian cystectomy was performed. Although paraovarian cysts are mostly asymptomatic, those arising near the fimbrial end can lead to torsion of the fallopian tube, therefore it should always be considered a possible cause of acute abdomen in adolescent girls with adnexal cysts on ultrasound. Timely surgical intervention can prevent complications such as fallopian tube necrosis, gangrene, removal of the tube, and its long-term implications especially in women of the reproductive age group.

## Introduction

Paraovarian cysts (POCs) also referred to as paratubal cyst or hydatid cyst of Morgagni, represent approximately 10% of adnexal masses [1]. Most of the time, they are asymptomatic but occasionally it can be complicated by torsion, hemorrhage and rupture, especially if large in size [2]. POCs can occur in any age group from neonate to menopause but are more commonly seen in the reproductive age groups between 21 and 40 years [3]. For diagnostic purposes, ultrasound can help in detecting adnexal masses and ovarian cyst accidents such as haemorrhage, but the mere presence of blood flow on Doppler ultrasound of the ovaries does not completely rule out ovarian torsion [3]. POCs are usually unilateral, thin-walled, unilocular and arise in the broad ligament. It may be difficult to reliably differentiate a POC from an ovarian cyst by imaging [4]. Therefore, they are often identified and removed surgically [5]. The condition is frequently misdiagnosed with acute appendicitis and ovarian cyst accidents such as ovarian torsion. The diagnosis in most of the cases is never established before laparoscopy. We present here a case of a fimbrial POC torsion, which presented as an adnexal cyst causing acute lower abdominal pain.

## Case presentation

A 19-year-old virgo intacta presented in the gynecology outpatient department complaining of severe dysmenorrhea of less than 24 hours duration and her menstrual cycle had started the same day. Pain located in the lower abdomen was acute in onset. It was associated with nausea and three to four episodes of vomiting. She had no urinary or bowel symptoms. The menstrual flow was normal and she had always had a regular menstrual cycle with no dysmenorrhea in the past. On examination, she appeared dehydrated, her pulse was 78 beats per minute but feeble, blood pressure was 110/70 mmHg and temperature was 36.5 degrees celsius. On abdominal examination, she had tenderness on deep palpation in the hypogastrium. There was no rebound tenderness, guarding or abdominal rigidity, and gut sounds were audible. On admission, urinalysis showed signs of infection with numerous pus cells, bacteria, nitrite and ketones were positive. However, other tests such as blood leucocyte count, hemoglobin, C-reactive protein levels were within normal limits.

**Figure 1:**
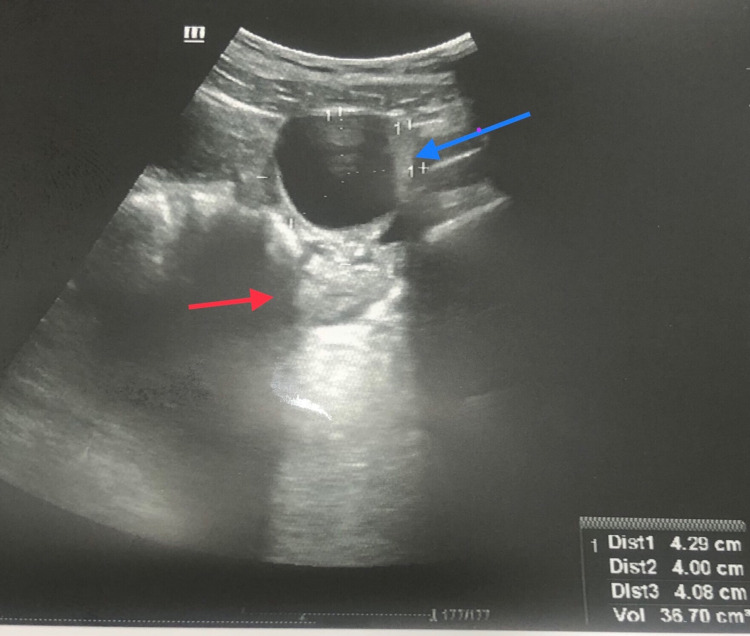
Ultrasound pelvis showing left paraovarian cyst (blue arrow) and left ovary visualized separately (red arrow).

Pelvic ultrasound showed a normal size anteverted uterus measuring 73 x 40 x 42 mm in size, with an endometrial thickness of 5 mm. There was a left POC measuring 30 mm in size and both ovaries were of normal size, which was separately visualized. Doppler study showed normal color and spectral flow in both ovaries making the possibility of ovarian torsion less likely (Figure 1).

**Figure 2:**
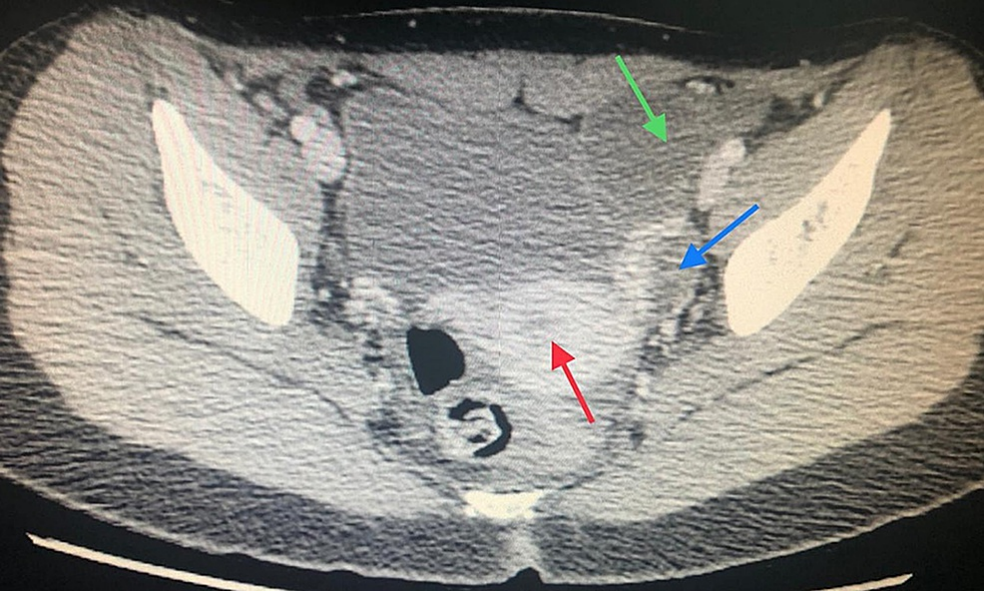
CT scan pelvis with contrast shows left paraovarian cyst (green arrow), uterus (red arrow) and left ovary can be visualized separately (blue arrow).

A CT scan pelvis with contrast was carried out which showed a well-defined unilocular POC of 34 mm in size in the left adnexa, with normal-looking ovaries and other pelvic organs including the appendix (Figure 2).

**Figure 3:**
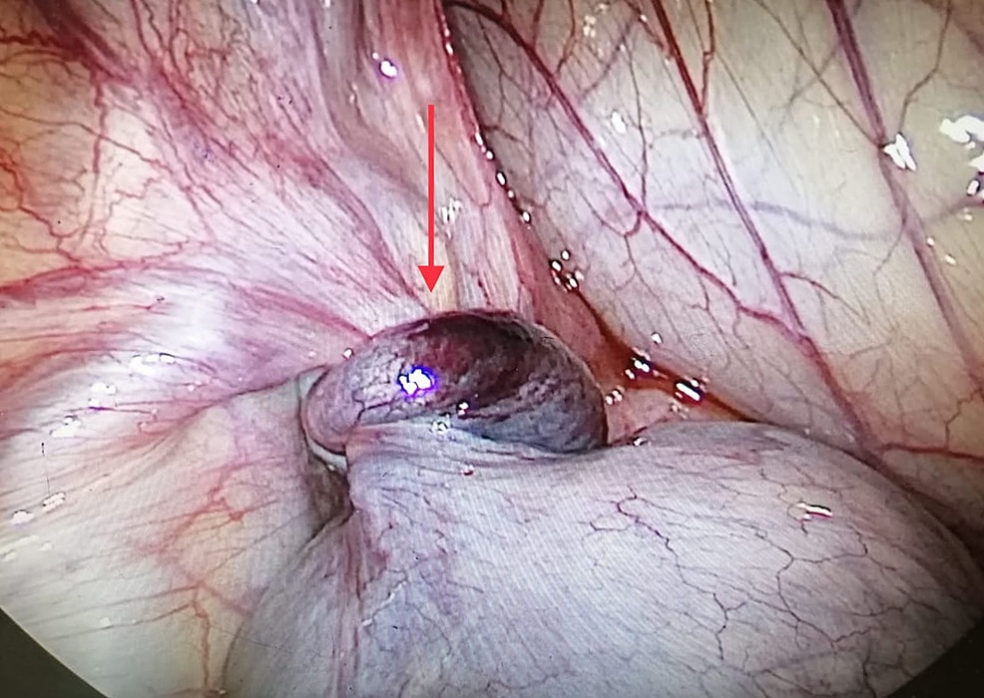
Left-sided paraovarian cyst with fallopian tube torsion (red arrow).

The empirical intravenous antibiotic was started in view of urinary tract infection, after sending urine for culture and sensitivity. Despite giving painkillers according to the WHO pain ladder, the pain persisted along with retching and vomiting six hours after the admission. A working diagnosis of POC accident (hemorrhage, rupture or torsion) was made and laparoscopy was performed. Laparoscopic findings were consistent with torsion of left fallopian tube with a unilocular fimbrial POC 4 cm in size approximately (Figure 3).

**Figure 4:**
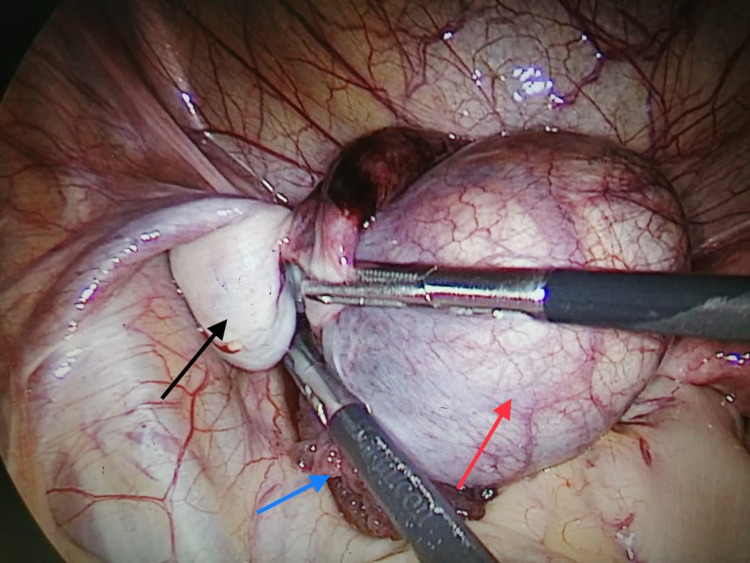
Left paraovarian fimbrial cyst (red arrow), ovary (black arrow) and fimbrial end of the left fallopian tube (blue arrow).

The fallopian tube was twisted once, it was detorted and a linear incision along the wall of the cyst was made with Ligasure® (Figure 4). The POC was completely enucleated intact with the help of Maryland grasper and a tissue sample was sent for histopathalogical examination. Even though there was a delay in diagnosis of eight hours, the fallopian tube wall throughout its entire length was healthy and there were no signs of necrosis or gangrene. It was therefore conserved. The uterus, right-sided tube, both ovaries, other pelvic and abdominal organs appeared normal. The patient had an uneventful postoperative recovery and was discharged the next morning. The histopathological report was consistent with the POC. The patient did well at the follow-up visit.

## Discussion

Fallopian tube torsion is a rare cause of severe pelvic pain and it is very difficult to diagnose preoperatively because of its less distinctive signs and symptoms. Unfortunately, there are no characteristic laboratory findings or radiological features that can help in differentiating this condition from others. Its incidence is estimated at one in 1,500,000 [6]. The available laboratory or imaging studies such as ultrasound pelvis, Doppler ultrasound, CT scan or MRI cannot confirm fallopian tube torsion. They can, however, rule out other abdominal and pelvic conditions with similar clinical characteristics, such as nephrolithiasis, cholelithiasis, appendicitis, extrauterine pregnancy, tubo-ovarian abscess and pancreatitis [6]. The possibility of ovarian torsion on the basis of the presence of blood flow on Doppler ultrasound can never completely be ruled out. CT scan can help in ruling out appendicular abscess, sigmoid colon abscess, tubo-ovarian abscess with its characteristic features but if present, MRI should be the preferred modality because of the low risk of ionizing radiation in women of reproductive age group.

The differential diagnosis of fallopian tube torsion includes acute appendicitis, ectopic pregnancy, pelvic inflammatory disease, twisted ovarian cyst, ovarian cyst rupture, ovarian cyst hemorrhage, hydrosalpinx, degenerative leiomyoma and acute diverticulitis [7]. Many reports indicate that torsion of the fallopian tube is more common on the right side than on the left [8].

There are no guidelines available for the management of fallopian tube torsion hitherto. However, due to its presentation as acute pelvic or abdominal pain, the definitive diagnosis mostly is made with diagnostic laparoscopy or exploratory laparotomy. Laparoscopy, being the gold standard, is preferable where expertise is available, and the patient is hemodynamically stable. An additional factor is that the size of the cyst should be operable, according to the Royal College of Obstetricians and Gynecologists (RCOG) guidelines on the management of ovarian cysts in premenopausal women, cysts greater than 7 cm in size leads to increased chances of cyst rupture at the time of surgery [9]. The benefits of laparoscopic surgery include lesser post-operative pain, febrile morbidity, quicker recovery, lesser cost and early discharge from the hospital. There is not enough evidence available regarding the effect of tubal function and fertility after detorsion [10]. With the available information, salpingectomy is the recommended treatment in cases with delayed gangrenous changes, whereas detorsion is recommended in cases without gangrenous changes [11]. The lack of specificity of signs and symptoms often fails to alert the physician to the possibility of fallopian tube torsion secondary to paraovarian or paratubal cyst torsion, making diagnosis difficult [12]. Although rare, the possibility of torsion of the fallopian tube should always be considered in young girls presenting with sudden abdominal pain, and an early surgical intervention preferably laparoscopy should be considered aiming at saving the tube [13].

## Conclusions

Fallopian tube torsion and fimbrial POCs may be a rare entity but it is one of the most important causes of acute abdominal or pelvic pain in women in the reproductive age group, especially with evidence of adnexal cyst on pelvic ultrasound.

Although less information is available so far regarding the long-term implications of salpingectomy on future fecundity, early surgical intervention in the form of detorsion of the fallopian tube and removal of the POC can prevent complications like necrosis, gangrene, rupture and the need for removal of the affected fallopian tube.
